# Search for an Association between V249I and T280M CX3CR1 Genetic Polymorphisms, Endothelial Injury and Preeclampsia: The ECLAXIR Study

**DOI:** 10.1371/journal.pone.0006192

**Published:** 2009-07-09

**Authors:** Alain Stepanian, Soraya Benchenni, Tiphaine Beillat-Lucas, Sophie Omnes, Fannie Defay, Edith Peynaud-Debayle, Gabriel Baron, Agnès Le Querrec, Michel Dreyfus, Laurence Salomon, Vassilis Tsatsaris, Dominique de Prost, Laurent Mandelbrot, for the ECLAXIR study group

**Affiliations:** 1 AP-HP, Hôpital Louis Mourier, Service d'Hématologie Biologique, Colombes, France; 2 CHU Clémenceau, Unité de Gynécologie-Obstétrique et Médecine de la Reproduction, Caen, France; 3 AP-HP, Hôpital Bichat-Claude-Bernard, Service de Gynécologie-Obstétrique, Paris, France; 4 AP-HP, Hôpital Bichat-Claude Bernard, Département d'Epidémiologie, Biostatistique et Recherche Clinique, Paris, France; 5 CHU Côte de Nacre, Service d'Hématologie Biologique, Caen, France; 6 AP-HP, Groupe Hospitalier Cochin Saint-Vincent de Paul, Service de Gynécologie-Obstétrique, Paris, France; 7 AP-HP, Hôpital Louis Mourier, Service de Gynécologie-Obstétrique, Colombes, France; 8 CIB Phénogen du GHU Nord, Paris, France; 9 Université Paris 7–Denis Diderot, Paris, France; 10 AP-HP, Hôpital Louis Mourier, Département de Santé Publique (Evaluation, Recherche Clinique, Information médicale, Vigilances)–(DIM), Colombes, France; Leiden University Medical Center, Netherlands

## Abstract

**Background:**

Preeclampsia and coronary-artery disease share risk factors, suggesting common pathophysiological mechanisms. CX3CR1/CX3CL1 mediates leukocyte migration and adhesion and has been implicated in the pathophysiology of several inflammatory diseases. M280/I249 variants of CX3CR1 are associated with an atheroprotective effect and reduced endothelial dysfunction. The aim of this study was to search for an association between V249I and T280M polymorphisms of CX3CR1, preeclampsia and endothelial dysfunction.

**Methodology/Principal Findings:**

We explored these polymorphisms with real-time polymerase chain reaction in a case-control study (184 white women with preeclampsia and 184 matched normotensive pregnant women). Endothelial dysfunction biomarkers including von Willebrand factor, VCAM-1 and thrombomodulin, as well as the soluble form of CX3CL1 were measured by enzyme-linked immunosorbent assays (ELISA). The I249 and M280 alleles were associated neither with preeclampsia, nor with its more severe form or with endothelial injury. In contrast, we found a trend toward increased CX3CL1 levels in preeclampsia patients, especially in early-onset- preeclampsia as compared to its level in later-onset- preeclampsia.

**Conclusions/Significance:**

This is the first study to characterize the CX3CR1 gene polymorphisms in patients with preeclampsia. We found no differences in genotype or haplotype frequencies between patients with PE and normal pregnancies, suggesting that maternal CX3CR1 V249I and T280M polymorphisms do not increase susceptibility to preeclampsia. Further studies should be performed to directly evaluate the pathophysiological role of CX3CL1, a molecule abundantly expressed in endometrium, which has been shown to stimulate human trophoblast migration.

## Introduction

Preeclampsia (PE) is a common complication occurring in about 2–7% of pregnancies, which is a major cause of perinatal and maternal mortality and morbidity [1]. As we gain insight into the pathophysiology of PE, placental ischemia and endothelial dysfunction stand out as key factors [2]. The increase of many markers of endothelial dysfunction, including thrombomodulin, von Willebrand factor (VWF) and VCAM-1, has been repeatedly reported in women with PE[2], [3]. It has been suggested that release of factors from the placenta in response to ischemia results in endothelial dysfunction throughout the maternal circulation. It also appears that the endothelial cell activation is reflective of a generally enhanced inflammatory state [4].

CX3CL1/fractalkine (CX3CL1) is a unique CX3C chemokine that is synthesized as a transmembrane molecule by endothelial cells activated by proinflammatory cytokines [5]. Soluble CX3CL1 can be released from the cell surface by proteolysis. The cell-surface bound molecule serves as a potent adhesion molecule while the secreted form has chemotactic activity. Its expression in endothelial cells mediates both migration and adhesion of CX3CR1-expressing cells [6]. CX3CR1, its 7-transmembrane domain G-protein-coupled receptor, is expressed on the surface of NK cells, monocytes and CD8+ T cells [6]. Recently, CX3CL1 and CX3CR1 were both found in endometrial cells [7]. Moreover, these authors also identified CX3CR1 expression on invading human trophoblasts [8] thus suggesting an involvement of these molecules in the control of the utero-placental vascularisation.

Two common single-nucleotide polymorphisms located in the coding sequence of the CX3CR1 gene have been described: they cause amino acid changes from valine to isoleucine at position 249 (V249I) and from threonine to methionine at position 280 (T280M) in the CX3CR1 protein [9]. These two polymorphisms are in strong linkage disequilibrium, forming a common I_249_M_280_ haplotype. Their association with several inflammatory diseases including arteriosclerosis and coronary artery disease has been demonstrated [10], [11], [12]. Interestingly, the IM haplotype was associated with a reduced risk of acute coronary events and atherosclerosis, and with a decreased endothelial reactivity. V249I and T280M involvement in these situations was explained by functional mutated CX3CR1 analysis showing impaired receptor expression and altered increased adhesive capacities [9], [10], [13].

Several studies have shown that women with a history of PE are at increased risk of cardiovascular complications later in life [14], [15]. In addition, PE and coronary-artery disease share many risk factors including hypertension, diabetes and dyslipidaemia [16], suggesting common pathophysiological mechanisms: besides impaired insulin sensitivity and inflammation, impaired microvascular function has been described [17], [18]. Finally, striking similarities were reported between the atherosclerotic plaque and atherosis lesions of spiral arteries in PE [19]. For these reasons, we hypothesized that genetic variations of CX3CR1 might be involved in PE as they are in coronary artery disease.

The aim of our study was to search for an association between V249I and T280M polymorphisms of CX3CR1, PE and endothelial dysfunction. We explored these polymorphisms in a case-control study and evaluated endothelial dysfunction by measuring several biomarkers including VWF, VCAM-1 and thrombomodulin, as well as the soluble form of CX3CL1.

## Materials and Methods

### Ethics Statement

Written informed consent to take blood samples was obtained from each woman before enrolment in our study which was approved by the Ethics Committee (Comité de Protection des Personnes dans la Recherche Biomédicale, CCPPRB) of Hôpital Bichat-Claude Bernard (Paris). Written parental consent was also obtained to take blood samples from newborns.

### Study design and subjects

The ECLAXIR study is a multicenter case-control study for which participants were recruited between May 2003 and October 2007 in 6 French university hospitals. The cases were 184 white women with PE. Inclusion criteria were age ≥18 years, Caucasian origin (defined as 4 grandparents born in Europe, or in the Maghreb), gestational age ≥20 weeks, and PE defined according to the definition of the American College of Obstetricians and Gynecologists (ACOG) [20]. Patients with proteinuria occurring before 20 gestational weeks were excluded. Controls were normotensive pregnant women; for each PE patient, a control matched for maternal age (±2 years), gestational term (±2 weeks) and ethnic origin (European or Maghrebian) was included. Controls who developed PE after enrolment were excluded from the study and replaced by another subject matched with the corresponding case.

PE was defined according to the ACOG [20] as elevated blood pressure (≥140/90 mm Hg) occurring after 20 weeks of gestation with previously normal blood pressure, and proteinuria (≥0.3 g in a 24-hour urine specimen).

Severe PE was defined according to the ACOG [20] as the presence of at least one of the following criteria: blood pressure ≥160/110 mm Hg in 2 measurements 4 hours apart while the patient is on bed rest; proteinuria ≥5 g in a 24-hour urine specimen; oliguria of less than 25 mL per hour; cerebral or visual disturbances; pulmonary oedema or cyanosis; epigastric pain; impaired liver function defined as serum aspartate aminotransferase concentrations (ASAT) ≥70 IU/L; thrombocytopenia defined as platelet count less than 100 Giga/L; fetal growth restriction defined as a Z-score lower than −1.88. This value calculated according to Salomon et al [21] is equivalent to a birth weight below the third percentile for gestational age.

HELLP syndrome (Haemolysis, Elevated Liver enzymes, Low Platelet count) was defined as the 3 following combined criteria haemolysis (lactic dehydrogenase>600 IU/L, decreased haptoglobin (<0.64 g/L) concentration or the presence of schizocytes in the peripheral blood), thrombocytopenia (platelet count<100 Giga/L) and ASAT>70 IU/L.

PE was defined as early if gestational age at diagnosis was <34 weeks.

### Blood sampling and DNA extraction

Venous blood was collected, at the time of enrolment, in case and control women into 1∶10 final volume of 3.8% sodium citrate and 15% K_3_EDTA solution. Platelet-poor plasma was obtained from citrated blood by centrifugation at 2500 g for 20 minutes, and aliquoted samples were stored at −80°C before being tested. DNA extraction was carried out with K_3_EDTA blood and QIAmp DNA Blood Midi Kit (QIAGEN, Courtaboeuf, France) according to the manufacturer's instructions.

At birth, cord blood was taken on K_3_EDTA in 182 newborns to allow identification of their genetic status.

### Screening for polymorphisms

The CX3CR1 gene V249I and T280M mutations were identified using a real time polymerase chain reaction method (PCR) [22] with slight modifications in the forward T280M primer (5′-AGGTGGTAAAGGTATCTTCTGAACTT-3′) and allele specific LNA Taqman probes: 5′-FAM-ACTG[+A][+G]A[+C]G[+G]T[+T]GCAT-TAMRA-3′ and 5′-TET-TGACTG[+A][+G]A[+T]G[+G]T[+T]GCAT-TAMRA-3′ probes for Tyr^280^ and Met^280^, respectively. Reactions were performed using the Rotor-Gene 3000 (Corbett Research) with slight modifications for V249I: 3 min at 50°C and 10 min at 95°C of initial denaturation, followed by 30 cycles with 20 sec at 95°C and 1 min at 64°C.

### Laboratory assays

ELISA was used to assess protein levels: von Willebrand factor antigen (Asserachrom® vWF:Ag, STAGO, France), VCAM-1 (Human VCAM-1/CD106, R&D Systems, USA), thrombomodulin (Asserachrom® thrombomodulin, STAGO, France) and soluble fractalkine (Human CX3CL1/Fracalkine, R&D Systems, USA). For the soluble fractalkine ELISA kit, the lower detection limit in plasma was 0.28 ng/mL with a coefficient of variability of 14.9% at 0.38 ng/mL.

### Statistics

Qualitative values are reported as percentages and quantitative values as mean±SD. Allelic frequencies were calculated by gene counting. Hardy-Weinberg equilibrium was tested by a χ^2^ test with 1 degree of freedom. Conditional logistic regression analysis performed with SAS was used to compare qualitative and quantitative variables between cases and controls; results were expressed as odds ratios (ORs) with 95% confidence intervals (CI)s. The relative contributions of V249I (VI+II versus VV) and T280M (TM+MM versus TT) genotypes and that of combined genotypes (versus the VV/TT reference combined genotype) to the occurrence of PE were estimated after accounting for known PE risk factors (nulliparity, pregestational BMI, pregestational diabetes, multiple pregnancy, personal history of PE or thrombosis, connective tissue disease or antiphospholipid antibody syndrome). An adjustment for the other polymorphism was also done (adjustment on M allele when evaluating V249I and adjustment on I allele when evaluating T280M). The Student's t test or Kruskal-Wallis test were used to compare quantitative variables among cases and controls. The program PHASE (version 2.1.1) was used to estimate frequencies of haplotypes [23]. The relative contribution of V249I and T280M genotypes to the severity and early onset of PE was estimated using conditional logistic regression analysis after accounting for the other polymorphism. The χ^2^ test was used to compare combined genotype and haplotype frequencies among cases according to the severity or the onset of PE. For comparisons of endothelial markers, a linear regression analysis was used to account for gestational age.

The prevalence of subjects carrying the I249 allele previously detected in our control population [10] was equal to 49%. With our sample size, the probability of detecting, at α = 0.05 (2-tailed test) an odds ratio of 0.55 for cases was 80%. The power of the study was obtained with the software SPSS SamplePower2.

## Results

### Study population

Between May 2003 and October 2007, 223 cases and 203 control patients were enrolled. Fourteen control patients were excluded for having developed PE after enrolment; five patients with PE were excluded because of Middle Eastern or Asian ethnic origins. Thus, 184 matched pairs of women were analysed.

Table 1 shows general demographic, obstetrical and medical characteristics of the study population. The 184 patients and controls were matched for maternal age, gestational age, and ethnic origin (141 pairs were from Europe, 40 pairs from the Maghreb and 3 pairs had mixed European/Maghrebian origins). Obesity (defined as a pre-pregnancy body mass index [BMI]>30 kg/m^2^) was more frequent in the cases than in controls (*p* = 0.001). As expected, nulliparity (75.4 *vs* 53.8%), multiple pregnancies (6.0 *vs* 0%), preterm delivery (68.9 *vs* 4.4%) and fetal growth retardation (12.5 *vs* 1.6%) were significantly more frequent in cases than in controls. Personal or familial history of PE was significantly more frequent in cases. We also found a greater proportion of preeclamptic women with a personal history of pregnancy loss before 22 weeks of gestation (20.3 *vs* 12.6%, *p*<0.03) or thrombosis (2.2 *vs* 0%; *p* = 0.04). In the latter group, 3 patients had a history of deep vein thrombosis and one of pulmonary embolism. By contrast, there were no statistically significant differences between cases and controls for pregestational diabetes, primipaternity, connective tissue disease or antiphospholipid antibody syndrome.

**Table 1:** Demographic, obstetrical and medical characteristics of the study population.

Characteristics	Cases (n = 184)	Controls (n = 184)	Odds ratio (95% Cl)	*p* value
**Age (years)** - mean±SD	31.1±6.0	31.2±5.5	-	-
**Ethnicity**				
European % (n)	76.6 (141)	76.6 (141)	-	-
Maghrebian % (n)	21.7 (40)	21.7 (40)	-	-
European/Maghrebian % (n)	1.6 (3)	1.6 (3)	-	-
**Gestational age (weeks) at enrolment** mean±SD	33.9±3.8	33.7±3.8	-	-
**BMI before pregnancy>30 kg/m^2^** % (n)	14.8 (27)	4.4 (8)	3.86 (1.68 to 8.86)	<0.01
**Nulliparity** % (n)	75.4 (138)	53.8 (99)	2.7 (1.67 to 4.35)	<0.01
**Primipaternity** % (n)	11.0 (20)	5.5 (10)	2.0 (0.090 to 4.45)	0.08
**Multiple pregnancy** % (n)	6.0 (11)	0	NA	<0.01
**Preterm delivery (<37 GW)** % (n)	68.9 (126)	4.4 (8)	59.5 (14.71 to 240.7)	<0.01
**Fetal growth restriction** % (n)	12.5 (24)	1.6 (3)	8.67 (2.56 to 29.31)	<0.01
**History of preeclampsia**				
Personal % (n)	6.6 (12)	0	NA	<0.01
Familial % (n)	10.9 (20)	3.3 (6)	3.8 (1.42 to 10.18)	<0.01
**History of venous thrombosis**				
Personal % (n)	2.2 (4)	0	NA	<0.05
Familial % (n)	10.3 (19)	5.4 (10)	2.1 (0.92 to 4.92)	0.08
**Previous pregnancy loss before 22 GW** % (n)	20.3 (37)	12.6 (23)	2.0 (1.08 to 3.72)	<0.03
**Previous fetal death after 22 GW** % (n)	1.1 (2)	1.7 (3)	0.7 (0.11 to 3.99)	0.65
**Pregestational diabetes** % (n)	2.8 (5)	0.6 (1)	4.99 (0.58 to 42.78)	0.14
**Connective tissue disease or APLS** % (n)	2.2 (4)	1.6 (3)	1.33 (0.30 to 5.96)	0.70

Some variation in number owing to missing data. BMI, body mass index; NA, not applicable; GW, gestation weeks; APLS, antiphospholipid syndrome. Fetal growth restriction <3d centile.

In the cases, 80.4% of women had severe PE and 46.2% had early-onset PE; 3.8% had a HELLP syndrome.

### Preeclampsia and CX3CR1 polymorphisms

The frequencies of the V249I and T280M polymorphisms showed no deviation from Hardy–Weinberg equilibrium. Moreover, no statistically significant difference in genotype frequencies was found between cases and controls. The adjusted odds ratios (ORs) associated with the I249 (VI+II versus VV genotype) and M280 (TM+MM versus TT genotype) were 1.10 (95% confidence interval [CI], 0.6–2.0; *p* = 0.75) and 1.41 (95% CI, 0.7–2.8; *p* = 0.33), respectively (Table 2). Further adjustment for the other polymorphism did not change the significance of the results (OR, 95% CI: 0.78, 0.3–1.8 [p = 0.56] and 1.79, 0.7–4.6, [p = 0.23], respectively). The V249I and T280M polymorphisms are in complete linkage disequilibrium and generate 6 combined genotypes of the 9 theoretically possible (Table 3) and only 3 haplotypes (V249T280, I249T280 and I249M280). Indeed, all subjects carrying allele M280 also carry allele I249, whereas in some subjects, allele I249 is associated with allele T280. The frequencies of combined genotypes and haplotypes were similar between subjects with and without PE (Table 3). In some cases, the genetic status of the newborns was identified. There was no statistically significant difference either in the genotype frequencies of the 2 polymorphisms in the newborns from PE (n = 71) or control mothers (n = 112) (data not shown). Moreover, in controls, the I and M allelic frequencies did not differ significantly between European and Maghrebian ethnic groups (0.24 *vs* 0.31 for I allele, and 0.13 *vs* 0.17 for M allele).

**Table 2:** Genotype frequencies of the V249I and T280M polymorphisms of the CX3CR1 gene in cases and controls.

		Cases	Controls	Odds ratio	95% Cl	*p* value
		(n = 184)	(n = 184)			
**V249I**	II % (n)	5.4 (10)	7.6 (14)	1.10*	0.60–2.03*	0.75*
	VI % (n)	40.8 (75)	37.0 (68)	0.78**	0.34–1.79**	0.56**
	VV % (n)	53.8 (99)	55.4 (102)	1		-
**T280M**	MM % (n)	1.1 (2)	2.7 (5)	1.41*	0.70–2.84*	0.33*
	TM % (n)	26.6 (49)	22.8 (42)	1.79**	0.69–4.61**	0.23**
	TT % (n)	72.3 (133)	74.5 (137)	1		-

* ORs (95% CI, p values) adjusted for PE risk factors, associated with the (II+VI) *versus* the VV genotype and with the (MM+TM) *versus* the TT genotype.

** ORs (95% CI, p values) further adjusted for the other polymorphism.

**Table 3:** Combined genotype and haplotype frequencies of the V249I and T280M polymorphisms of the CX3CR1 gene in cases and controls.

			Cases (n = 184)	Controls (n = 184)	
**Combined genotype**	**V249I**	**T280M**	**% (n)**	**% (n)**	**Adjusted OR (95% CI)** *
	VV	TT	53.8 (99)	55.4 (102)	1
	VI	TT	17.9 (33)	17.4 (32)	0.8 (0.4–1.8)
	VI	TM	22.8 (42)	19.6 (36)	1.6 (0.7–3.4)
	II	TT	0.5 (1)	1.6 (3)	NA
	II	MM	1.1 (2)	2.7 (5)	NA
	II	TM	3.8 (7)	3.3 (6)	0.9 (0.2–4.7)
**Haplotype**			**%**	**%**	**OR (95% CI)**
	VT		74.2	73.9	1
	IT		11.4	11.9	0.9 (0.6–1.5)
	IM		14.4	14.1	1.0 (0.7–1.5)

Values are percentages of subjects (number) or haplotypes. NA not analysed because of small number of subjects (n≤3 per group).

* ORs adjusted for PE risk factors.

We then searched for an association between CX3CR1 genotypes and the severity of PE. Table 4 shows the frequencies of the two CX3CR1 tested polymorphisms in the case group, as a function of PE severity or time of onset. We did not find any association of CX3CR1 V249I or T280M polymorphisms with severe forms of PE or early-onset PE. Similar results were obtained when using combined genotypes or haplotypes (Table 4).

**Table 4:** Genotype, combined genotype and haplotype frequencies of the V249I and T280M polymorphisms of the CX3CR1 gene in the preeclampsia group, distributed according to severity and time of onset.

			Severe PE	NS PE	OR*	p	Early PE	Late PE	OR*	p
			(n = 148)	(n = 36)	(95% CI)		(n = 85)	(n = 99)	(95% CI)	
**Genotype**	**V249I**	II % (n)	5.4 (8)	5.6 (2)			7.1 (6)	4.0 (4)		
		VI % (n)	43.2 (64)	30.6 (11)	3.1 (0.9–11.2)	0.08	45.9 (39)	36.4 (36)	1.9 (0.8–4.1)	0.12
		VV % (n)	51.4 (76)	63.9 (23)	1		47.1 (40)	59.6 (59)	1	
	**T280M**	MM % (n)	0.7 (1)	2.8 (1)			1.2 (1)	1.0 (1)		
		TM % (n)	27.0 (40)	25.0 (9)	0.4 (0.1–1.6)	0.19	29.4 (25)	24.2 (24)	0.8 (0.3–1.9)	0.66
		TT % (n)	72.3 (107)	72.2 (26)	1		69.4 (59)	74.8 (74)	1	
**Combined genotype**		VI/TT % (n)	20.3 (30)	8.3 (3)		0.21†	21.2 (18)	15.2 (15)		0.29†
		VI/TM % (n)	23.0 (34)	22.2 (8)			24.7 (21)	21.2 (21)		
		VV/TT % (n)	51.4 (76)	63.9 (23)			47.1 (40)	59.6 (59)		
**Haplotype**		IT %	12.8	5.6		0.22$	14.1	9.1		0.20$
		IM %	14.2	15.3			15.9	13.1		
		VT %	73.0	79.2			70.0	77.8		

Values are the percentage of subjects (number) and haplotypes. PE, preeclampsia. NS, non severe.

* ORs associated with the (II+VI) *versus* the VV genotype and with the (MM+TM) *versus* the TT genotype are adjusted for the other polymorphism.

† Chi^2^ comparison for the 3 common combined genotypes.

$ Chi^2^ comparison for the 3 haplotypes.

### Endothelial dysfunction

Table 5 shows mean values of endothelial lesion markers and of soluble CX3CL1 that were measured in both groups. Correlation between concentrations of these proteins and gestational age are shown in Table 6. The plasma levels of VWF and VCAM-1 were significantly higher in women with PE than in paired controls (p<10^−3^) as was thrombomodulin (*p* = 0.05). Soluble CX3CL1 levels were also increased in cases versus controls but this did not reach statistical significance (*p* = 0.08). Interestingly, the CX3CL1 levels did not vary with gestational age in the control group, but showed a tendency to decrease in the PE group (*p* = 0.05). Moreover, CX3CL1 levels tended to be higher in patients with very early-onset PE beginning at 25–29 weeks as compared with controls analysed at the same gestational age (Table 6).

**Table 5:** Mean maternal plasma concentrations of VWF:Ag, Thrombomodulin, VCAM-1 and CX3CL1 in paired cases and controls.

	n	Cases	n	Controls	Odds ratio (95% Cl)	*p*
**VWF:Ag (IU/dL)**	157	363.1±133.1	157	222.0±92.7	1.13 (1.08–1.17)	<10^−3^
**Thrombomodulin (ng/mL)**	157	62.5±29.4	157	54.4±42.5	1.1 (1.00–1.21)	0.05
**VCAM-1 (ng/mL)**	156	1156.9±430.5	156	647.4±188.3	1.06 (1.04–1.08)	<10^−3^
**CX3CL1 (ng/mL)**	152	1.1±2.1	152	0.7±0.9	10.22 (0.78–134.03)	0.08

Values are mean±SD. Some variation in numbers due to missing data. VWF:Ag, von Willebrand factor antigen; VCAM-1, soluble vascular cell adhesion molecule-1; CX3CL1 (soluble form). Odds ratios are calculated for 10 units of each parameter.

**Table 6:** Laboratory marker concentrations according to gestational age at enrolment.

				Gestational age*			
		25 to 29		30 to 34		35 to 41	*p*
**PREECLAMPSIA CASES**	**n**		**n**		**n**		
VWF:Ag (IU/dL)	31	355.5±116.1	70	379.5±134.1	59	349.6±136.1	0.35
Thrombomodulin (ng/mL)	31	58.4±25.3	70	61.3±29.2	59	65.0±31.3	0.56
VCAM-1 (ng/mL)	31	1212.5±446.3	69	1142.3±445.8	59	1140.3±398.4	0.72
CX3CL1 (ng/mL)	31	1.7±3.8	68	1.1±1.6	58	0.7±0.6	0.05
**CONTROLS**	**n**		**n**		**n**		
VWF:Ag (IU/dL)	38	176.3±58.6	72	205.9±80.8	68	260.6±97.8	<10^−3^
Thrombomodulin (ng/mL)	38	42.8±17.7	72	57.0±58.4	68	56.6±20.1	<10^−3^
VCAM-1 (ng/mL)	37	592.1±158.3	72	612.7±177.7	68	704.9±186.0	<10^−3^
CX3CL1 (ng/mL)	38	0.5±0.4	72	0.7±1.3	68	0.7±0.6	0.27

Enrolment was done at the onset of preeclampsia for cases, and at matched gestational age for controls.

Values are mean±SD. VWF:Ag, von Willebrand factor antigen; VCAM-1, soluble vascular cell adhesion molecule-1.

* gestational age is given in weeks.

In order to search for an association between these laboratory markers of endothelial injury and CX3CR1 genotypes, we compared their mean values according to genotypes in the control and the case groups. Our results showed no difference in the concentration of these markers according to the V249I and T280M genotypes in both groups of women (data not shown). Moreover, there was no difference according, to the severity of PE or to the gestational age at onset (data not shown).

## Discussion

Our results strongly suggest that the occurrence of PE is independent of CX3CR1 V249I or T280M polymorphisms of the mother. Thus, despite several pathophysiological similarities and risk factors between atherosclerosis and PE, we did not confirm our hypothesis of a protective effect of genetic CX3CR1 polymorphisms in PE. The I249 and M280 alleles were associated neither with PE, nor with its more severe form, nor with endothelial injury evaluated by 3 laboratory markers.

Since the familial nature of PE has been known for many years, various linkage and candidate gene studies have been carried out, as recently reviewed by Mütze *et al* [24]. More than 50 candidate genes have been studied, with 8 genes accounting for two thirds of the studies [25]. Genome-wide searches identified several loci suggestive of linkage but with inconsistent results. Recently, a heterogeneity-based genome search meta-analysis identified genetic regions (bins) associated with PE or severe PE [26]. In severe PE, one of these bins was located in the 3q11.1-3q21.2 region of chromosome 3, within the large cluster of chemokine receptors [27], [28]. Our study is the first to evaluate the role of the CX3CR1 gene.

Although the cause of preeclampsia remains unknown, three main aetiological factors are involved, namely immune maladaptation, placental ischemia and increased oxidative stress [29]. Studies of the last two decades also showed that PE is a multisystemic syndrome notably involving vasoconstriction, endothelial dysfunction and increased inflammatory response [29]. Endothelial dysfunction and inflammation are also involved in the development of atherosclerosis. CX3CR1/CX3CL1 mediates a mechanism of leukocyte capture, firm adhesion, and activation which is independent of that induced by integrins and has been implicated in the pathophysiology of several inflammatory diseases, including renal diseases [30], allograft rejection [31], and atherogenesis [32]. Several studies reported the atheroprotective effect of M280/I249 variants [10], [11], although the specific role of each variant was difficult to elucidate due to the strong linkage disequilibrium between the two polymorphisms. The function of CX3CR1 in atherosclerosis was further confirmed by studies showing that inactivating the CX3CR1 gene reduced the atherosclerotic lesion area as compared with wild mice in the apoe−/− background [33]. Interestingly, the peripheral mononuclear blood cells of individuals with the CX3CR1 IM haplotype adhered more strongly to membrane-CX3CL1 than did those of CX3CR1-VT donors [13], thus suggesting that this excess adhesion to CX3CL1-expressing endothelial cells might diminish the extravasation of monocytes across the endothelial barrier. In another study, binding experiments with peripheral mononuclear blood cells from healthy donors showed that cells from heterozygous VI individual expressed fewer CX3CR1 receptors at the cell surface [10]. Although this hypothesis is attractive, we did not find a molecular basis to support this mechanism, as PE was not associated with the genetic polymorphisms whether considered separately or as combined genotypes or haplotypes. We did not find either an association between the VI/II or TM/MM genotypes and the more severe form of PE (Table 4) or with the degree of endothelial lesion measured using 3 well-known markers of endothelial injury. Indeed, these marker levels were quite similar according to genotype in cases as in controls. We also studied the distribution of genotypes between the two distinct forms of PE, the early-onset, placental and the late-onset, maternal form [29]. We found no significant difference in genotype or haplotype frequencies between these 2 forms. Ethnic groups of cases and controls were carefully matched to remove this frequent confounder.

In this study, we report for the first time soluble CX3CL1 levels in normal and PE pregnancies. We found higher plasma concentration of soluble CX3CL1 in cases versus controls at diagnosis, although the difference fell short of statistical significance. Moreover, CX3CL1 levels were higher in very early-onset PE diagnosed between 25 and 29 weeks of gestational age as compared to PE of later onset. These results however should be considered with caution due to the large coefficient of variation of the CX3CL1 assay.

The membrane-anchored form of CX3CL1 is primarily expressed on the inflamed endothelium; recent work showed the presence of its soluble form in the amniotic fluid of pregnant women [34]. CX3CL1 is also abundant in entire endometrium and placenta while its receptor (CX3CR1), is expressed on trophoblast cell lines [8]. Human trophoblast migration was shown to occur in response to CX3CL1 at the foetal-maternal interface [8], [35]. Interestingly, the mechanisms of embryo-endometrial apposition/adhesion/invasion appear to be analogous to those of leukocyte-endothelium rolling/adhesion/extravasation, as discussed by Dominguez *et al* [36]. The increase in soluble CX3CL1 which we observed in our study is a consequence of more proteolysis of the membrane-anchored form. Whether this results in less functional cellular CX3CL1 and less adhesion of trophoblast cells to spiral arteries remains to be investigated. Interestingly, a recent study showed that CX3CL1 confers an essential survival signal to monocytes which might provide a mechanistic explanation for its role in atherogenesis [37]. Our results thus suggest the possible involvement in PE of CX3CL1 alterations (rather than CX3CR1). Alternatively, increased CX3CL1 may represent a positive response of the endometrium to placental ischemia. Indeed, CX3CL1 has been shown to have pro-angiogenic properties [38] which may counteract the deleterious anti-angiogenic effects of soluble flt1 and endoglin [39].

The recently identified expression of CX3CR1 by trophoblast cells further suggests the importance of studying the fetal (or paternal) genotype. Because cord blood was not always collected, the number of cases and controls with fetal DNA was insufficient to conclude in the present study.

In conclusion, this is the first study to characterize the CX3CR1 gene polymorphisms in patients with PE. We found no differences in the allele or genotype frequencies between patients with PE and normal pregnancies. Our study suggests that the CX3CR1 V249I and T280M polymorphisms are not associated with the development of PE in white women. In contrast, our results may suggest a pathophysiological role for CX3CL1, a molecule abundantly expressed in endometrium and recently shown to be a survival signal for monocytes.
